# Towards a nordic digital education in hospital at home: a group-interview study

**DOI:** 10.1186/s12909-026-10739-0

**Published:** 2026-10-31

**Authors:** Jeanette Johansson, Gunilla Carlsson, Agneta Malmgren Fänge, Helene Åvik Persson

**Affiliations:** Department of Health Sciences, Faculty of Medicine, Lund University, Lund, Sweden

**Keywords:** Challenges, Digital care, Expectation, International collaboration, Sustainable education, Thematic analysis, Vision

## Abstract

**Background:**

Hospital at Home (HaH) is an increasingly common healthcare model nationally and internationally, and consequently there is a need for digital health education in the area, including in the Nordic countries. Even in similar contexts, cross-national collaboration remains complex because cultural variations shape communication, decision-making and perceptions of healthcare priorities. However, knowledge of how this unfolds in cross-national projects remains limited. Thus, the aim of this study was to explore visions, expectations and challenges among members of a consortium involved in the development of a Nordic digital health education in Hospital at Home, HaH.

**Methods:**

A qualitative design with group interviews was applied, with data collected at three different occasions during December 2023 to September 2024. A total of 18 participants from the NorDigHE project consortium were included. Data was analyzed using the RITA process.

**Results:**

The results contain one theme From vision to sustainable reality – education for the future, and comprises four subthemes: *Managing cross-national differences in healthcare and healthcare education; Managing diverse professional and cultural perspectives;* C*reating an attractive learning context*, and; C*reating a valid and sustainable content.* The results reflect upon the importance of being responsive to cross-national differences, language barriers regarding diverse perspectives, practical strategies for creating an education comprising different learning activities and on the challenge of developing an education for healthcare professionals with the possibility to evolve with technology.

**Conclusions:**

Cross-national collaboration, in our study developing a cross-national digital health education on HaH, seems to create both challenges and rewarding experiences among the consortium members. It is apparent that it is important to understand the aspects of diversity that can arise within a cross-national collaboration, and to create an open and responsive environment between the members to maintain a solid foundation for an effective process towards the shared goal despite differences in healthcare systems and in professional and cultural perspectives. Our study has the potential to serve as a guidance on how to prepare for future partnerships to avoid loss of efficiency and effectiveness in cross-national consortia. The findings can also be used to improve the outcomes of cross-national projects, by approaching the collaboration with cultural intelligence.

**Supplementary Information:**

The online version contains supplementary material available at https://doi.org/10.1186/s12909-026-10739-0.

## Background

The landscape of healthcare is currently undergoing a paradigm shift, with hospital level treatment in the patient’s own home, Hospital at Home (HaH), as an increasingly common model of care [1] nationally and internationally. HaH strives to merge home and hospital for improved patient care [2, 3] and encompasses a broad range of healthcare services all provided around the clock in the patients’ own homes [4]. Despite the growing use of HaH, variation remains in how the model of care is defined [5]. Leff et al. [5] highlighted the need for developing a definition of HaH to establish a common understanding of the model. In 2023, the World Hospital at Home Community presented an international consensus definition of HaH [6] as “Hospital at Home/ Hospital in the Home (HaH/ HITH) is an acute clinical service that takes staff, equipment, technologies, medication and skills usually provided in hospitals and delivers that hospital care to selected people in their homes or in nursing homes. It substitutes for acute inpatient hospital care. It’s goal is to improve the lives of sick people who need hospitals, by changing the culture of hospitals to deliver hospital-level care at home” [6].

The Nordic countries cooperate extensively in healthcare, and there is a high degree of exchange of patients, healthcare professionals and students in medicine and health. Despite many similarities among the countries, HaH is applied in diverse healthcare systems and cultural contexts. These conditions highlight the need for cross-national education endeavors that are responsive to these variations and can accommodate the different needs [1, 7, 8]. However, there is still a lack of knowledge of how the different needs and preferences play out in cross-national, education development projects.

To develop an education having the potential to reach a broad audience within individual countries and across borders [9, 10], the involvement of interprofessional teams with different backgrounds in the development process is necessary as it makes the education more successful [11], and potentially increases the validity of it [12]. Cross-Nordic collaboration is complex, as cultural variations influence communication styles, decision-making processes, and perceptions of healthcare priorities [7]. Despite this diversity in needs, it is important that members of projects targeting education development share the same visions, expectations and goals [13, 14]. That is, project success is shaped by multiple factors, first by accounting for the project members’ different perspectives, secondly, the type and context of the project. Within a project the short- and long-term outcomes may differ, and project members play a central role in defining, creating, and distributing content along the process, and the members contribute to achieving alignment in decision making during project initiation, planning and implementation [13]. This involvement can contribute to the defining of project requirements, identifying resources and supporting the development process throughout the project [14], while organizational design and collaboration across multiple actors further influence the achievement of the project’s goal [13]. Over time, projects often become more complex due to changes in content requirements, abandonment of allocated work tasks by the different partners and upcoming technical challenges [15]. Therefore, projects need to be monitored throughout to make sure that necessary adjustments to process and content are accommodated to [13, 16]. This includes exploring the different perspectives that exist among those involved in the development process. However, there is still a lack of knowledge of how the different perspectives, needs and preferences play out in cross-national, education development projects. Therefore, the aim of this study was to explore visions, expectations and challenges over time among members of a consortium involved in the development of a Nordic digital health education in HaH.

## Methods

### Study design

A qualitative approach was employed with group interviews conducted over time aiming at following the program development process. The study is reported in line with the Consolidated Criteria for Reporting Qualitative Research (COREQ) [17].

### Study context

The study is part of the larger project “Nordic Digital Health and Education (NorDigHE)”, consisting of a consortium of public and private actors from Denmark, Norway and Sweden, including hospitals and higher education institutions (HEI), and small and medium enterprises (SME). The project aims to develop and evaluate a digital education targeting health professionals, ensuring the competence to deliver hospital care in the home using digital technology. The education is equivalent to 5 credits at the second cycle level and aims to be a fully online student-centered education [18], including the following topics: provision of hospital care at home, communication and strategies to support patient participation, e-health competence, digital technologies as a tool in care, systematic quality work and patient safety.

### Recruitment and participants

Initially, the authors, all members of the consortium, informed the NorDigHE consortium partners about the study, its aim, and the structure of the interviews. Those who wished to take part were invited to contact the last author (HÅP). In total, 18 participants where included, see Table 2. Only one consortium member took part at all three interview occasions, eleven took part at two occasions (five on the first and second occasion, five at the second and third occasion and one at the first and third occasion) and six consortium members took part only at one occasion (three at the first and three at the third).

### Data collection

We collected data by means of semi-structured group interviews. The interview guide was developed based on the domains in the NASSS (**N**on-adoption and **A**bandonment of technologies by individuals and the challenges to **S**cale-up, **S**pread and **S**ustainability of such technology in health and care organizations) framework [19], comprising seven domains: Condition (health issue addressed), Technology (digital tools used), Value Proposition (perceived benefits), Adopters (individuals or groups adopting the innovation), Organization (structures and culture within healthcare settings), Wider System (the broader healthcare environment), and Embedding and Adaptation Over Time (long-term integration and evolution of the innovation) [20, 21]. The pedagogical structure applied in the education was developed before the first interviews, and at the time of the third interview occasion, a pilot version of each education module existed. Before each interview occasion the participants were divided into two groups, each with as much diversity as possible in professional background and organizational affiliation. All partners within the consortium were represented at each interview occasion but not in each group. The moderator (HÅP) started each interview by explaining its purpose, followed by three broad questions targeting previous experience of educational projects, and of challenges and opportunities within this project. Thereafter, the interview proceeded according to the interview guide (supplementary file 1). Probing questions was used. All interviews were conducted in Scandinavian languages with all participants speaking their native language. A second researcher observed and took notes. All interviews were recorded and lasted between 41 and 50 min. All interviews were conducted at regular, physical NorDigHE consortium meetings, in December 2023, and March and September 2024 respectively.

### Data analysis

Data from the interviews were analyzed using the audio recordings, and the RITA procedure (Rapid Identification of Themes from Audio recordings) was applied [22], complemented by thematic analysis, inspired by Braun and Clarke [23]. RITA is a method that allows the researcher to rapidly create themes by coding, using the audio recordings instead of more time-consuming transcription. The RITA procedure involves a five-steps process, where the first step involves specifying our research foci, in our case visions, expectations and challenges [22]. In the second step, two of the authors, JJ and HÅP, individually familiarized themselves with the data by repeatedly listening to the audio recordings. JJ and HÅP then started coding according to the identified foci. A codebook was created by listening to the first two interviews, noting observations within the discussions, focusing on visions, expectations and challenges, and subsequently naming the initial codes. JJ and HÅP listened to the interviews and independently coded the data. Thereafter, JJ and HÅP analyzed and coded all interviews by listening to the audio recordings and comparing them with the coded material from the first two audio recordings. Additional segments of interest were identified, and codes created were added. Initially, the interviews from each occasion were analyzed separately, and in the later stage they were analyzed as one dataset. In the third step of the RITA procedure, a codebook was created. In the fourth step, the codebook was tested and refined through an iterative process, resulting in 68 identified codes. Finally, in the fifth step, all data were coded using the revised codebook. Iteratively, four subthemes were developed, and reviewed in relation to the study aim. Subthemes and themes reflecting the aim of the study where integrated. AMF and GC independently reviewed the codes, subthemes, and themes. JJ again listened to the recordings and marked quotes of interest. Along the process, disagreements were solved in an iterative process involving all authors and by returning to the audio recordings. For examples of the analytical process, see Table 1.

**Table 1:** Examples of the analytical process

Observations in data/discussions	Open coding	Code	Subtheme	Theme
We do not agree with each other completely on which didactics we should use. Are films something to use and why in that case? Do we need to consider learning outcomes that need students’ ability to draw from their own practice, or is it something we can deliver by including different patient cases? What do we think about e-learning? It is something we need to agree on when we create simulation via our private partners, but what does simulation mean within e-learning?	Challenges in didactics, what to include in the education. Which learning activities should be prioritized. Is there an agreement concerning learning activities?	Didactics and the use of different learning outcomes within the education.	Creating an attractive learning context	From vision to sustainable reality - education for the future

## Results

In total, 18 participants, six men and 12 women participated, whereas five from Swedish partners, nine from Danish partners and four from Norwegian partners (Table 2).

**Table 2:** Sociodemographic characteristics of participants for each group interview occasion (*N* = 18)

Characteristics	Interview 1		Interview 2		Interview 3	
	**Group 1a (*n*** **= 5)**	**Group 1b (*n*** **= 5)**	**Group 2a (*n*** **= 5)**	**Group 2b (*n*** **= 6)**	**Group 3a*n*** **= 5)**	**Group 3b (*n*** **= 5)**
Gender, *n*						
Female/Male	4/1	5/0	3/2	6/0	3/2	3/2
Organization, *n*						
Hospital	1	1	1	2	2	2
SME*	1	1	1		1	
HIE**	3	3	3	4	2	3
Partner country, *n*						
Sweden	3	2	1	1	1	
Norway	1	1	1	2	1	1
Denmark	1	2	3	3	3	4
Profession, *n*						
Reg. professional w PhD	1	0	1	0	0	1
Reg. professional w/o PhD	2	3	1	4	2	2
Scientist*** w PhD	1	1	2	1	3	0
Other w/o PhD****	1	1	1	1	0	2

*SME  small and medium enterprises

**HEI Higher Education Institution

***Scientist = Anthropology, Education/Pedagogy, Physiology, Public health

****Other = Graphic design, Public health, Systems development

**Fig. 1:**
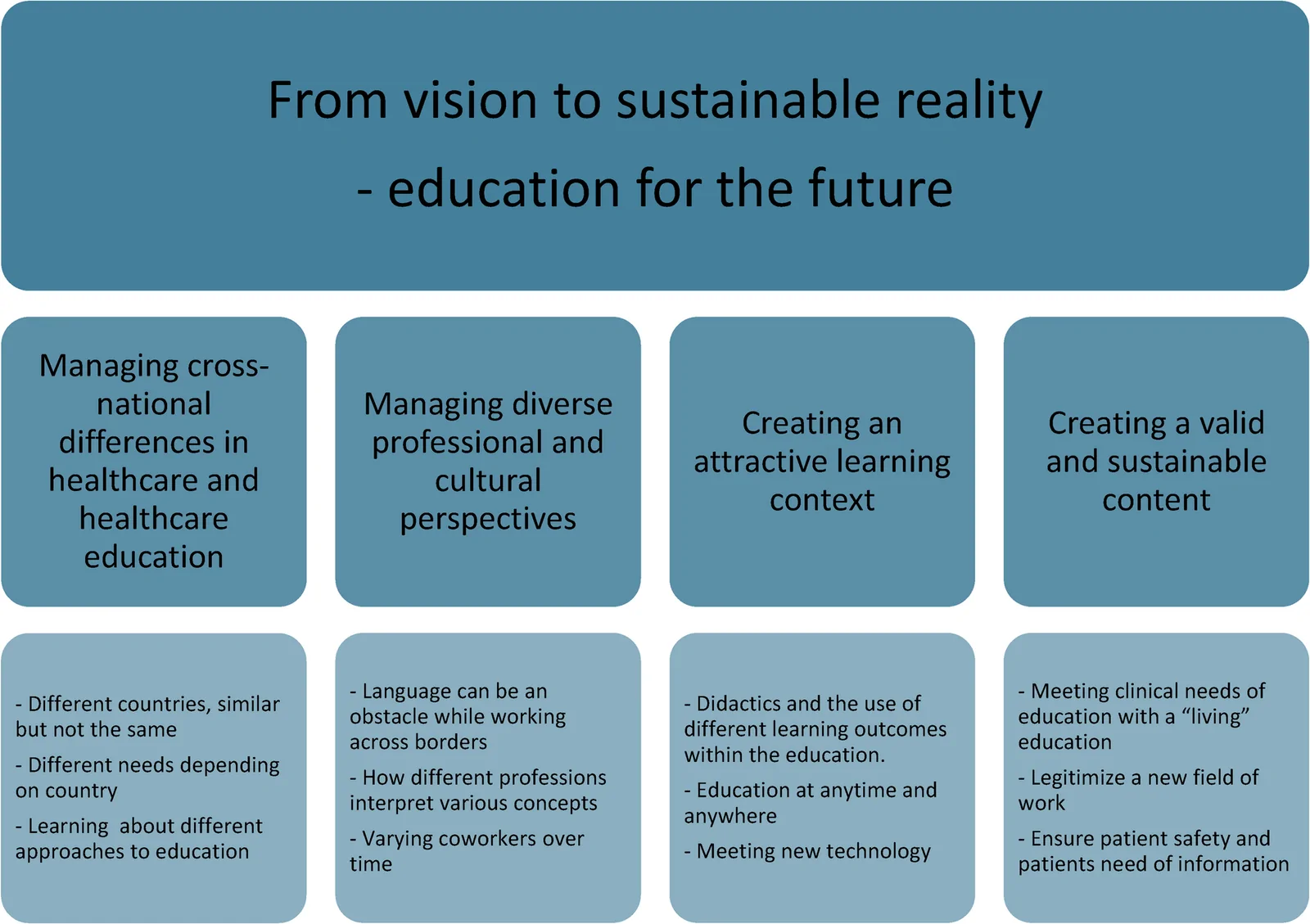
Overview of identified theme and subthemes, including examples of codes

One theme emerged: *From vision to sustainable reality – education for the future* comprising four subthemes (Fig. 1).

### From vision to sustainable reality – education for the future

The theme reflects the challenges with cross-national differences and emphasizes the importance of being responsive to such differences when creating a mutual path forward. It addresses diverse perspectives and how they were dealt with along the project. The theme also comprises reflections and considerations related to creating the education. The theme comprises four subthemes, *managing cross-national differences in healthcare and healthcare education*,* managing diverse professional and cultural perspectives*, *creating an attractive learning context* and *creating a valid and sustainable content.*

#### Managing cross-national differences in healthcare and healthcare education

Even though Denmark, Norway and Sweden share similarities, the participants expressed that both the clinical and educational settings differ. The difference in systems was most prominent at the early stages of the project process, but by the end of it, due to the consortium’s accommodation to differences across countries, the participants were hopeful of an increased transferability in the education to other contexts.

“For me, I think it is as you somewhat mentioned, to put together three Nordic countries which all of them deliver very good care but now have to find a kind of a sync in how to do this from now on, together – I see very many opportunities in it.” (Interview 1).

The participants described that healthcare settings differ across countries, particularly the extent to which healthcare professionals from the hospital care for patients in their homes, from the majority being physical visits to only having contacts digitally. To accommodate these differences, the participants over time adjusted the content of the education to come up with an education suitable for HaH in a Nordic context. The participants emphasized that all consortium members have an equal responsibility to develop an education that meets cross-national needs. Different plans for delivering the HaH education in each country were revealed, including plans to e.g., offer the course within educational programs in the first cycle or as a separate course in the second cycle.

#### Managing diverse professional and cultural perspectives

The participants were challenged by the diversity in perspectives, e.g., those created by differences in professional backgrounds and competences within the consortium. The participants emphasized the importance of listening to one another and respecting the diverse perspectives, knowledge, and cultural backgrounds. Also, concepts used to describe national contexts varied, and there were challenges added by having three languages, creating discussions and at some points misunderstandings, which created confusion. This respect for linguistic and cultural diversity played a key role in the decisions related to the education development process.

“We come from different sectors, I come from the education sector. It is a strength but we…. Think we all agree that there is a need, it’s also a collective power. We totally agree on that… We just need to agree a bit more on the language…. The differences” (Interview 3).

It was time consuming to overcome the differences, however, despite this, the participants expressed that belonging to a cross-national consortium was personally rewarding. Being creative and open resulted in a positive working climate, and by the third interview occasion the participants expressed that the consortium had come to a mutual understanding of their differences in perspectives and concepts.

“The communication has been difficult, lack of communication language wise, I don’t think we have talked about that enough, but it is a major factor in relation to how we understand each other. It is a strength, but it is also what makes it complicated.” (Interview 5).

In addition, the consortium members changed over time and new members needed to be updated, adding new perspectives, which at times caused frustration. Over time, the participants expressed how they became more comfortable with the project, yet they remained focused on their own tasks and responsibilities. Later, the participants described the teams as cohesive, both in terms of collaboration and in the education development process.

#### Creating an attractive learning context

The participants discussed how the education can set the agenda for tomorrow’s education and healthcare within HaH, and considered the project to be right on time. The education aims to attract all healthcare professionals, and educational matters were discussed extensively.

The participants believed that by including different learning activities, the digital education could attract and keep the healthcare professionals’ interest, and thus, create a motivating learning context. The participants expressed different ways of digital learning and how to include e.g. games, simulations, videos, reflective questions and quizzes.

According to the participants the education should evolve over time to meet the changing landscape of healthcare demands and adaptation to the constantly developing technology, at the same time having high quality and being highly competitive. The participants recognized the flexibility of digital education as both beneficial and empowering, since it allows healthcare professionals to access the course independently of time and place. This flexibility was seen as invaluable, especially when workplaces may not always provide time off for traditional education.

“It is flexible and it is digital, you can take it whenever, I think that is an advantage. That we clearly think through that there will a progression to demonstrate that they have reached the learning outcomes. But I think the flexibility is important.” (Interview 2).

The participants emphasized the importance of advertising the education to make it appealing for healthcare professionals and to encourage managers to allocate time for their staff to take the course.

#### Creating a valid and sustainable content

The participants expressed expectations that by developing an education related to HaH, healthcare professionals could be equipped with skills for e.g., fostering confidence and ensuring patient safety, necessary for high quality acute care at home. The fact that patients may have a broader health concern beyond primary issues was also discussed. Accordingly, healthcare professionals require a wide range of knowledge across different areas of healthcare. They expressed an ambition to develop an education that can help to provide high quality care but also raised questions about how to make others see the value of it.

In order to expand the healthcare professionals’ knowledge and motivation for the education, and at the same time increasing the credibility of it, the importance of creating patient cases and of integrating different clinical elements in the education was highlighted.

“…but also to motivate healthcare professionals that it [the education] is connected to the reality they come from … it is a challenge for the clinicians, they want to work with cases that are connected to them… a challenge when you create cases that they send a signal that it is not directly targeted to them…” (Interview 4).

Also mentioned was the possibility for hospitals to screen patients eligible for HaH. The participants emphasized that it is not the diagnosis of the patient that is important to identify and address in the education, but more the patients’ resources.

“We really haven’t talked about who is the target group, diagnostically.... that’s the patient’s diagnosis, is not the target group, rather the form of care...We have discussed a lot who the patient can be... it is good that it is more generally, that makes it more applicable since we don’t know what care will look like in the future....” (Interview 6)

## Discussion

In this qualitative interview study, the findings provide new knowledge into cross-national collaboration along the process of developing a Nordic digital health education. The theme, From vision to sustainable reality – developing an education meeting the future healthcare landscape, comprises four subthemes: Managing cross-national differences in healthcare and healthcare education; Managing diverse professional and cultural perspectives; Creating an attractive learning context; and; Creating a valid and sustainable content.

It was obvious from our findings that differences across countries could be challenging. That is, even though Scandinavian countries are similar in many ways, they also differ in context of health care, education and societal organization. Research to investigate collaboration in higher education has emphasized significant aspects for successful collaboration such as equal partnership and mutual respect for differences. At the same time, balancing ambitions with conditions was highlighted as another strategy for success with cross-national collaboration in education [24]. To maintain partnership in cross-border collaboration, Porcaro and Carrier’s [25] ten principles could be of use in the development of online modules. The principles include clarifying roles and responsibilities and building trust between the various parties involved in cross-national work. It is important to keep these perspectives in mind throughout the entire education development process [25]. Parmentier [26] reports that a co-design process of an education involving collaboration among teams from different nationalities requires time and relationship-building. The context of care, education and organization plays a crucial role in cross-border collaborations, which in turn makes it important to address diverse views of knowledge, perspectives concerning knowledge systems, and societal priorities that can influence the partnership [26]. Acknowledging and addressing cross-national differences and creating strategies for reflecting values, ethics and definitions of knowledge can help overcome national barriers, and thereby potentially strengthening the foundation for more sustainable and constructive collaborative processes.

Another finding from our study is the necessity of addressing different perspectives within cross-national collaboration. Cultural differences regarding both professional and educational backgrounds, as well as native languages, can create conceptual confusion, potentially slowing down the process and add frustration. Both in terms of understanding cross-national collaboration and different perspectives, considering cultural intelligence is important. According to Waterval [27] cultural intelligence means avoiding judging a particular experience until sufficient information is available, to adequately understand the situation and act accordingly. In particular, it is important to develop when members are striving for a greater understanding of cross-national collaboration, in order to shape experiences characterized by a higher degree of cooperation [27]. Similarly, Zolfaghari and Madjdi [28] found that a multidimensional view of culture is required, considering various aspects that influence values, such as nationality, profession and age, to develop trusting and co-creative groups. Moreover, ensuring that cultural competence is integrated into the collaboration is essential due to the understanding of different sociocultural contexts [29].

Research has also shown that it is essential to consider the linguistic aspects [30]. Linguistic differences within teams, may affect power dynamics and create imbalances in influence and trust between members. This can lead to deterioration of certain work processes, which in turn can affect members’ ability to utilize their formal position and expertise [30]. In addition, the study by Tudor Car and colleagues [31] found that educators who were co-designing faced challenges regarding communication between different professions but also between different nationalities. This is reinforced by previous research on psychological safety in teams [32], which means that participants should feel free to express their opinions, which in turn can promote openness and innovation, particularly necessary in complex development projects. That is, in addition to culture being one aspect to consider, recognizing linguistic differences between coworkers could help create a collaborative environment reducing the risks of conceptual confusion, hypothetically establishing a constructive process with less frustration due to misunderstandings.

Our findings also showed that, from the very beginning, the consortium expressed expectations to create an attractive, sustainable and high-quality education, with respect to both learning context and content. This preference has been taken into consideration by developing a completely digital education and is played out by the flexibility but remains to be evaluated. The advantage of flexibility as discussed by Hunck et al. [33], who reported an increased attendance when students were able to participate at any time or place. Hertling et al. [34], also highlighted that medical students viewed digitalization positively and flexibly, largely preparing them for practice. This is similar to findings reported by Prosen and Liĉen [12], that accessibility and flexibility can promote learning. However, reflections were also raised about the possible disadvantages of the flexibility of digital education, such as difficulties in getting to know the student group and the loss of discussions after lectures [34]. Ma et al. [35] emphasize the need for initiatives to increase digital health education in order to ensure that current and future healthcare professionals remain up to date and meet expected requirements. A European study [36] that examined medical students’ perceptions of digital health education revealed a demand for more education in digital health and how to guide patients in using digital health technology, which can apply to HaH care. They viewed digitalization in healthcare positively and wanted to play an active role, particularly regarding patients’ digital health literacy [37]. Developing a digital health education concerning HaH care could attract more clinicians as students and as well as motivate them to attend all elements of the education could also strengthen their comfort and provide them with the arguments to give care digitally.

Finally, this study addresses the aspects of creating a digital health education that is both valid and sustainable and that appeals in terms of both context and content. Prosen and Liĉen [12] highlight the ongoing transformation in the education sector, where digital teaching is being integrated and developed to a greater extent. Part of this shift involves creating valid education, using elements such as quizzes and videos, as well as simulations and virtual reality to create a more dynamic and interactive learning environment. Tharalson et al. [37] claims that adaptive learning platforms including simulations and videos can help students in their learning. This is consistent with the findings of this study, which emphasized the importance of incorporating various learning activities, such as games, simulations, and quizzes, that are relevant to everyday HaH practice. Ogundiya et al. [38] reflect similar findings, using several learning activities contributes to develop the student’s knowledge in a positive way and gives opportunity to meet different students’ needs of education. Furthermore, according to Poncette et al. [39] students and/or clinicians require an element of learning by doing and by including games and simulations in the education this could provide practical experience of digital care. However, digital transformation requires more than technical development to adapt and maintain digital teaching methods and didactic approaches. Developing digital skills and critical thinking to promote students for complex clinical practice [40] and a conscious design of learning activities that promotes student engagement and makes education appealing, promotes strategies to face challenges in digital transformation and to create sustainable pedagogy [12, 41]. Education is a facilitator for working with the Sustainable Development Goals (SDGs) in Agenda 2030, which is a central aspect at all levels of education today [42, 43]. In this work, digital competence and education are integrated with sustainable development and contribute to achieving sustainability goals [44, 45]. Developing a valid and sustainable digital health education with a variety of learning activities that are aligned with technology and focusing on HaH care could potentially contribute to meeting the transformational needs of healthcare sector and lead to HaH becoming a subspecialty in the future. In addition, healthcare practice and research are developing and the content of the education must be kept up to date.

### Strengths and limitations

This study has both strengths and limitations that can affect its trustworthiness, i.e., credibility, dependability, confirmability and transferability [46].

All data was collected through group interviews. Group interviews stimulate reflection and generate data through interaction between participants but the group setting may have influenced participants’ willingness to openly share challenges, disagreements and negative experiences within the project. This has most likely affected the study´s credibility by limiting the range of perspectives captured [47]. To reduce the risk of social desirability bias [48], efforts were made to create a safe environment where everyone could be heard. The participants were encouraged to share all perspectives, including critical reflections, as these were considered valuable. The moderator asked open-ended questions and actively encouraged participants to share a variety of experiences, both positive and negative. Participants were given opportunity to elaborate on or refine their answers without being challenged. In addition, the moderator in the interviews in this study has extensive experience in conducting group interviews and tried to balance the discussion by making sure that everyone had the possibility to speak freely [46].

Furthermore, the interviews were conducted in three different languages. Despite their similarity there are also considerable differences between them, making the interviews challenging at times. However, the participants facilitated the discussions by e.g. repeating statements or asking for clarification when they didn’t understand. A strength could be the fact that it was the same interviewer on all three occasions which increased dependability by enhancing trust, nuances of data and reduced variation in the process [46, 49].

Using the NASSS framework to create the interview guide supported the development of questions relevant to digital healthcare interventions [19], thus potentially strengthening the dependability of the study [46]. The interviews were analyzed using the RITA process for coding and identification of themes and sub-themes [22]. Using rapid analysis methods such as the RITA process was a deliberate choice due to the need for a quicker but still systematic analytic process and according to Vindrola-Padros and Johnson [50] rapid approaches can support time-efficient identification of themes while safeguarding the analytical quality, especially within health research contexts. Long et al. [51] report similar results; even if audio-coding was experienced as more difficult, it could be cost-efficient and it does present an equal result as coding with transcripts. Limitations associated with this approach can be the absence of transcription, which may reduce the level of detail and transparency of the analytical process, however, using the RITA process can strengthen the credibility of the results by creating an opportunity for the authors to capture the atmosphere within the discussions, such as tone, emphasis and pauses, while coding [22, 50]. Even if rapid methods can be perceived as less in-depth, prior research suggests that rapid methods can produce findings equal to more time-consuming methods using transcription, when applied systematically [50]. There is a possibility of focusing on the most noticeable data, which could lead to a lack of detail and to limit this risk the authors have repeatedly returned to the audio recordings. Member checks were also applied, involving 25% of the participants. Member check is a crucial tool that validates the accuracy and transparency of data and promotes collaboration between the researcher and participants [44], thus increasing the credibility of the findings [45]. Given the amount of data generated member checks were considered an adequate part of the process, and together with quotes from each interview the confirmability of the findings was strengthened [46]. All authors were involved in the analysis and had different professional backgrounds (occupational therapists, anesthetists nurse and public health nurse). The interpretations were discussed to challenge assumptions and enhance reflexivity. The first author conducted the initial analysis and was not involved in the project from the start. One co-author had been involved from the start, while remaining co-authors became involved in the project during an early phase. This brings out a mixed level of familiarity and involvement among the authors, which can be considered as a strength and supports the reflexivity in the analysis [52].

As a result of changes in the consortium’s composition early on, participants differed between interview occasions. Thus, different consortium members were interviewed at different occasions and in different group constellations. This could have affected the credibility of our findings due to the participants’ varying levels of experience of the project process. On the contrary, the group interviews took place over time which means the process was followed and reflected upon at each interview, potentially improving the credibility of the findings [46, 47]. Moreover, conducting a study that includes three different cultural contexts can increase the transferability of the results.

### Implications for practice

We make the following suggestions to teams setting out to conduct cross-national projects:

- Create strategies for reflecting values, ethics and definitions of knowledge early in the process, to maintain trust among the members of the team.
- Acknowledge and address cross-national differences already from the start of a collaboration project.
- Address cultural differences in the team, such as professional and educational backgrounds and native languages, and implement cultural intelligence strategies.
- Develop and implement strategies for promoting smooth onboarding of new team members.

## Conclusions

To conclude, cross-national collaboration, in our study developing a cross-national digital health education on HaH, seems to create both challenges and rewarding experiences among the consortium members. It is apparent that it is important to create an open and responsive environment between the members to maintain a solid foundation for an effective process towards the shared goal, despite differences in healthcare systems and education, and in professional and cultural perspectives. Our study has the potential to serve as a guidance on how to prepare for future partnerships to avoid loss of efficiency and effectiveness in cross-national consortia. The study findings can also be used to improve the outcomes of cross-national projects, especially by approaching the collaboration with cultural intelligence.

## Supplementary Information

Supplementary Material 1.

## Data Availability

The dataset generated and analyzed during the current study is not publicly available due to the risk of identifying participants and preserving anonymity.

## Abbreviations

HaH: Hospital at home
HEI: Higher education institutions
SME: Small and medium enterprises
