# Supplementary material for: Towards a nordic digital education in hospital at home: a group-interview study
Source: BMC Med Educ. 2026 Oct 31;26:1761. doi: 10.1186/s12909-026-10739-0 (PMC13723135; doi:10.1186/s12909-026-10739-0)
Supplement: Supplementary file 1 [file 12909_2026_10739_MOESM1_ESM.docx]

**Interview guide - NorDigHE**

- Present the aim and topic of the interview.
- Ask the participants to elaborate on their answers and to provide thick descriptions and examples.

Opening questions – to introduce the topic for forthcoming discussions and facilitate the participant’s reflection on the topic.

- What are your previous experiences of educational projects?
- Which possibilities or options do you find in this project, where a digital education is to be developed?
- What are the challenges you find in this project, where a digital education is to be developed?

Main Question (according to NASSS)

- What are the factors we need to consider when developing the education, regarding the patients’ medical condition and the hospital-at-home context?
- Clinical relevance, form of organization, different patient categories
- Coordination of several caregivers
- Which are the resources needed for dissemination of the digital education?
- Implementation
- Work processes
- What do you think the potential value of this education would be for the healthcare organizations?
- Usability/usefulness
- Costs
- What do you think the responses will be from the potential users of the digital education?
- Perceived readiness
- Expected consequences
- Are the organizations ready for implementation of the education?
- Traditions of change
- Support of pilot tests
- What are the external contextual possibilities and facilitators in the healthcare organizations
- What are the external contextual challenges in the healthcare organizations
- Technical and structural components and the implementation itself
- Competing, parallel development projects – how to deal with this?
- How do you consider the future of the education in terms of ”adaptation over time”
- Dependence on other actors
- Control of resources

Follow-up questions

- Clarification of uncertainties – Can you tell me more about/elaborate on this
- Did I understand you correctly when you said that…
- Do you have anything more to add?

Concluding questions

- Out of the questions we have discussed during the interview, which ones do you consider most important and why so?
- Is this an adequate summary? The summary question that is asked after the moderator/assistant moderator summarized the interview.
- Is there anything we should have talked about that which has not come up for discussion?
